# Bilateral congenital absence of the anterior cruciate ligament associated with bilateral knee and hip osteoarthritis: Case report

**DOI:** 10.1016/j.ijscr.2020.05.099

**Published:** 2020-06-12

**Authors:** Kwang-kyoun Kim, Tae-hyeong Kim, Dae-young Kim, Jae-kyu Choi

**Affiliations:** Department of Orthopaedic Surgery, Konyang Unversity Hospital, Gasoowon-dong, Seo-gu, Daejeon, 3536, Republic of Korea

**Keywords:** Knee instability, Congenital aplasia of cruciate ligament, Osteoarthritis, Case report

## Abstract

**Introduction:**

Congenital absence of the cruciate ligaments is very rare condition. Its association with congenital abnormalities of the spine, knee and hip, has been rarely reported. Most of the case reports were mostly reported before adolescence, so reports of the progression of this congenital disease at old age were extremely rare.

**Presentation of case:**

A 65-year-old woman had a 7-year history of both knee and hip pain. On physical examination, valgus and varus instability on both knees and painful limitation of motion in both hip joints were observed. Radiography and magnetic resonance imaging showed the absence of the anterior cruciate ligament with osteoarthritis on both knees and hip osteoarthritis. Total knee and hip arthroplasties were performed on both knee joints and total hip arthroplasty. The pain and instability of both knees and hips were lost for follow up of more than a year, and the patient is doing well in daily life.

**Discussion:**

Congenital absence of cruciate ligament may progress to osteoarthritis, do early detection and proper management is needed. In the treatment of older aged patients. Reports of treatment for this disorder are also very rare in older age. In old age, arthroplasty can be a useful treatment when the severe osteoarthritis is accompanied by instability.

**Conclusion:**

Since the congenital absence of the ACL in old age could lead to hip and knee osteoarthritis, periodic follow-up is necessary and arthroplasty can be a useful treatment when the severe osteoarthritis is accompanied by instability.

## Highlights

• Reports of the progression of congenital absence of cruciate ligament were very rare in old age.
• Authors reported a case of congenital absence of cruciate ligament with severe knee instability, knee and hip osteoarthritis.
• Congenital absence of cruciate ligament may progress to osteoarthritis, so early detection and proper management is needed.
• Total knee arthroplasty is useful for the treatment of arthritis and instability following congenital absence of cruciate ligament.

## 1 Introduction

Congenital absence of the cruciate ligament (CL) is a very rare anomaly. This condition has a prevalence of 0.017 per 1000 live births [1]. Normal development of the cruciate ligaments originates from the articular interzone around 7–10 weeks of gestational age during fetal development with the posterior cruciate ligament (PCL) forming first, followed by the anterior cruciate ligament [2]. This condition may be associated with other embryologic abnormalities of the lower limb, such as shortness of the femur and hypoplasia of the intercondylar tibial eminence, the intercondylar notch, and discoid meniscus [[3], [4], [5]]. Its association with congenital abnormalities in the hip and spine has been rarely reported. Since most of the reports have been in children before adolescence, reports of the progression and treatment of these abnormalities in older aged patients are even rarer. The authors experienced a patient with a congenital absence of the ACL with osteoarthritis in both knee joints and hip joints and achieved good results through total joint arthroplasty of the hips and knees. Herein, I report this case with a relevant literature review.

“Written informed consent was obtained from the patient for publication of this case report and the accompanying images.”

## 2 Case report

The patient was an unemployed 65-year-old female patient with height and weight of 158 cm and 48 kg, respectively. Her nationality and race were Korean and Mongoloid, respectively. She visited the outpatient clinic for pain in her knee and hip joints on both sides. The knee pain had developed seven years ago and was more severe on the right than on the left and caused a lot of disruption in her daily life. The patient also had been experiencing knee instability since adolescence, but no special diagnosis or management had been performed. The instability was aggravated by sitting, standing up or pivoting motions. The patient complained of a 3-year history of pain in the hip joints of both sides without any special trauma, which made it difficult to walk more than 500 m without walking aids. The patient had no past or family history of diseases that would be accompanied by multiple joint pain.

Upon physical examination, the right knee presented with slight joint effusion and no marked anterior or posterior laxity (negative Lachman and pivot shift tests and anterior/posterior drawer tests), but the patient had an instability on the frontal plane (laxity at the valgus: grade 3, laxity at the valgus; grade 3). The left knee also showed no marked anterior or posterior laxity (negative Lachman and pivot shift tests and anterior/posterior drawer tests), but instability on the frontal plane (laxity at the valgus: grade 3, laxity at the valgus; grade 3) was demonstrated. There was no limitation in the range of motion of either knee. Both hip joints were positive on the Patrick test and rolling test and both joints showed a limited range of motion (right: flexion 90°, extension 10°, abduction 25°, adduction 15°, internal rotation 10°, external rotation 20°; left: flexion 95°, extension 10°, abduction 20°, adduction 15°, internal rotation 15°, external rotation 20°).

Blood tests showed that the whole blood cell test, as well as the liver, kidney, thyroid, parathyroid, and adrenal function tests, were all within normal limits. Tests related to autoimmune diseases (rheumatoid factor, antinuclear antibody, anti-cyclic citrullinated peptide, and HLA B27) were all negative.

**Fig. 1:**
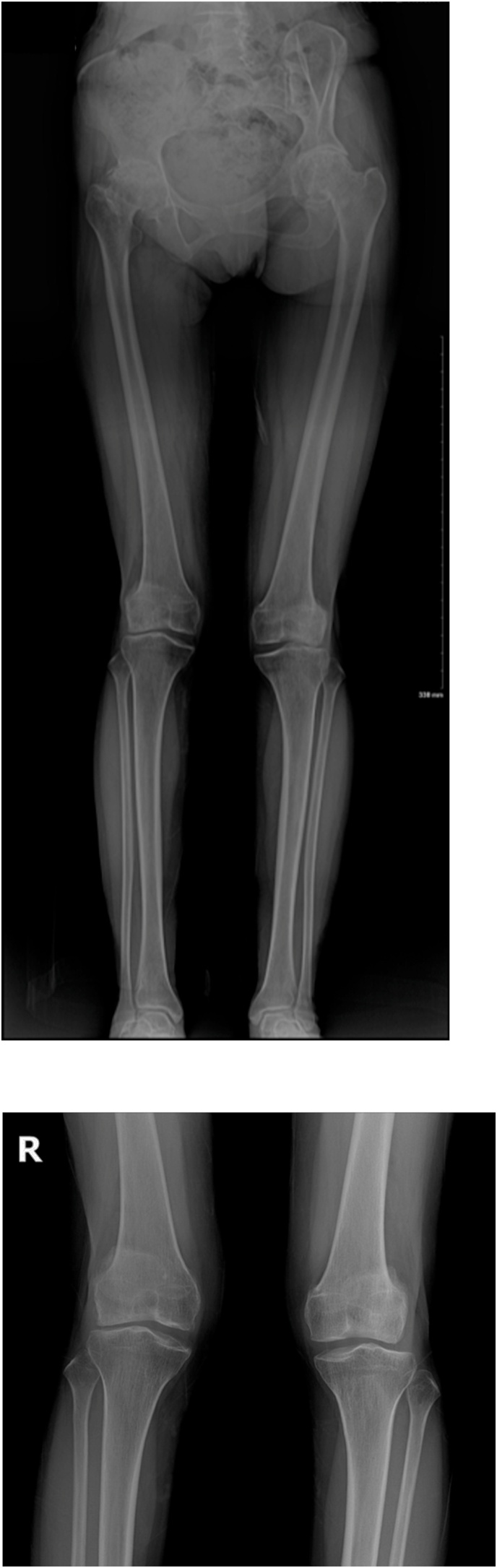
Radiography of preoperative lower extremity. A: Lower extremity scanogram showed more valgus anatomic axes (right: 12°, left 10°) than normal range (6°±3°). B: Osteoarthritis of both knees (Kellgren-Lawrence Grade III) are shown on lateral compartment and the tibial intercondylar eminence was absent in both knees. Both the medial and lateral condyles of the bilateral femurs as well as the femoral intercondylar notch were poorly developed.

**Fig. 2:**
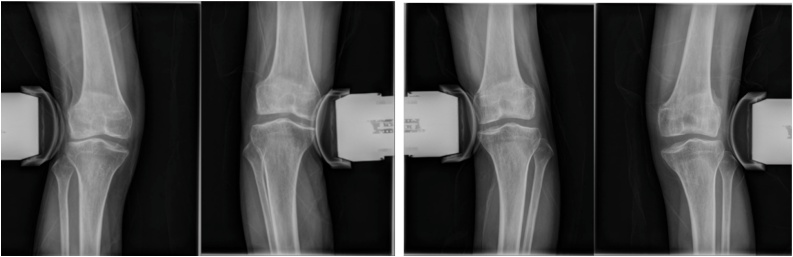
Physician-applied varus/valgus stress radiographs reveals lateral and medial compartment joint opening on the both knee and it indicated instability of both knees. A: Right knee. B: left knee.

**Fig. 3:**
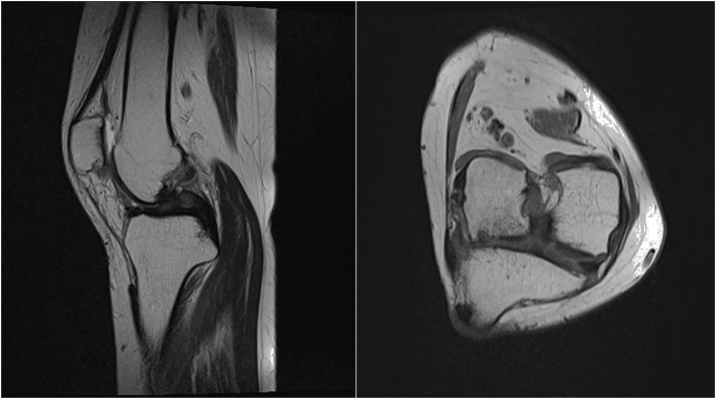
There was no identifiable structure with the recognizable features of the anterior cruciate ligament. A : T2 weighted sagittal MR image. B: T2 weighted axial MR image.

**Fig. 4:**
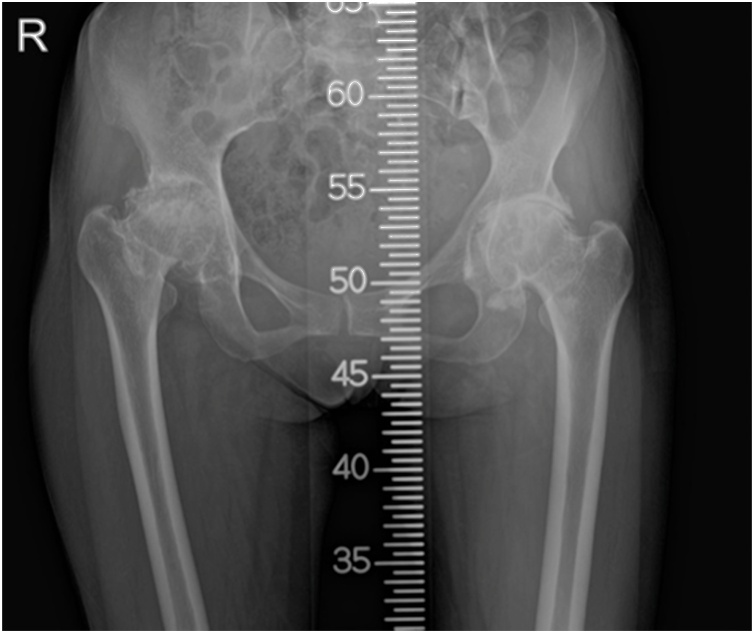
Radiography of both hips shows joint space loss, osteophyte formation, cyst formation and subchondral sclerosis.

A lower extremity scannogram showed more valgus anatomic axes (right: 12°, left: 10°) than normal (reference value: 6° ± 3°) (Fig. 1A). Both knee standing radiographs showed joint space narrowing of the lateral compartment with osteoarthritis (right: Kellgren-Lawrence grade 3, left: Kellgren-Lawrence grade 3) and hypoplasia of the tibial intercondylar eminence and flat trochlea femoralis were also seen (Fig. 1B). Physician-applied varus/valgus stress radiographs revealed lateral and medial compartment joint openings in both knees (Fig. 2). Magnetic resonance imaging (MRI) showed reduction of the cartilage thickness in the weight-bearing area of the lateral femoral condyle, a scarcely pronounced intercondylar notch and absence of the ACL (Fig. 3). On both hip anteroposterior views, both hip joints showed severe osteoarthritis (right: K–L grade 4, left: K–L grade 4) (Fig. 4).

On the basis of the above clinical and image findings, we diagnosed congenital aplasia of the ACL with secondary knee and hip osteoarthritis.

**Fig. 5:**
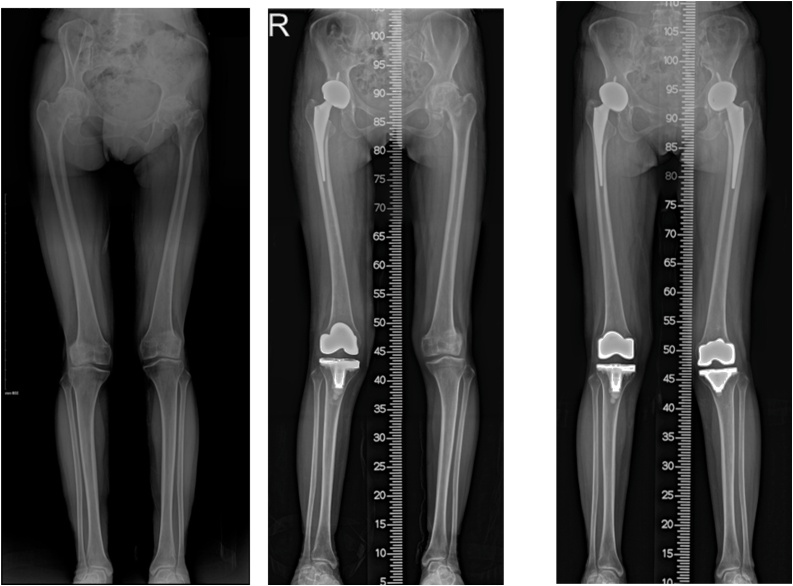
right hip and knee arthroplasty was performed, and in one year later, left hip and knee arthroplasty was implemented. A: Pre-operative lower extremity scanogram. The pelvis is tilted to the right and osteoarthritis changes were observed on both sides of the hips and knees. B: Post-operative lower extremity scanogram of total joint arthroplasty of the right knee and hip. After total arthroplasty of right hip and knee, the pelvis which had been tilted to the right was tilted slightly to the left then and valgus deformity was corrected when compared to the other side. C: Post-operative lower extremity scanogram of the left total knee and hip arthroplasty. After surgery on both sides, the pelvis of both sides became balanced and the leg length was also almost no different.

The patient complained of more pain in the right knee and right hip than in the left side, so right total knee and hip arthroplasty (TKA, THA) were performed simultaneously. About one year after the last surgery, the patient presented with more aggravated pain and instability of the left knee and hip joint. She underwent TKR and THR simultaneously (Fig. 5). As a result of the surgeries, the patient's symptoms improved and she was satisfied with the results. After the surgeries and follow-up for more than a year (right 24 months, left 12 months), both her knee and hip were pain-free without evidence of instability on physical examination.

## 3 Discussion

Since the first report by Niebauer and King in 1960, several cases of the congenital absence of the ACL have been reported [6]. Manner et al. classified the radiologic characteristics of 31 patients into three types in 2006 [1]. Type I was a group of patients that showed hypoplasic or aplasic of ACLs with a partially closed femoral notch and a hypoplastic tibial spine. In type II, the ACL aplasia was accompanied by hypoplasia of the PCL. In addition, the femoral notch and the tibial spine are worse than those in type I. Type III was classified as aplasia in both the ACL and PLC with the complete absence of the femoral intercondylar notch and aplasia of both tibial spines. The aforementioned classification did not include the accompanying presence or degree of osteoarthritis since most congenital absences of ACL were found before adolescence. However, based on this case, the degree of osteoarthritis and instability affected the choice of treatment method in old age, so a new classification is needed to take these factors into account.

In this case, the patient had severe hip osteoarthritis, which has never been reported in patients with a congenital absence of the ACL. The authors suggest that the associated abnormalities of hip dysplasia or hip dislocation had progressed to osteoarthritis in the congenital absence of the ACL [[7], [8], [9]]. If hip pathologic findings, such as hip dysplasia and congenital dislocation, are overlooked at a young age and untreated, they will proceed to severe osteoarthritis. Therefore, it is necessary to evaluate hip pathology in older patients with a congenital absence of the ACL and to be aware of the possibility of knee pathology in patients with hip osteoarthritis.

Treatment for the congenital absence of the ACL remains an open debate and the opinions vary between conservative and surgical treatments. The treatment of congenital aplasia of that ACL that occurs before adult age is determined depending on the degree of skeletal maturity and knee instability. Often, this malformation is well tolerated and, therefore, there is little need for surgery in childhood. Furthermore, it is often preferable to wait until skeletal maturity before proposing ligamentoplasty to prevent immature epiphysiodesis. Conservative treatment for congenital aplasia in the ACL can result in poor outcomes, as shown in a few reports [[10], [11], [12]]. Other authors have reported favorable results with cruciate ligament reconstruction [[13], [14], [15]]. Anterior instability foretells meniscal and chondral lesions and leads to early osteoarthritic degeneration of the knee. Another reason of operation is reducing physical activities in children is difficult to observe and is difficult for children accept it.

Cases of congenital aplasia of the ACL in old age are very rare and reports of treatment for the disorder are also very rare [[16], [17], [18]]. In the treatment of older aged patients, Frikha et al. reported two cases of valgus high tibial osteotomy (HTO) and one case of total knee arthroplasty [19]. However, in high tibial osteotomy, conjoined ligamentous instability with osteoarthritis has been associated with poorer HTO outcomes in some studies [[20], [21], [22]]. The authors achieved pain control and joint stability through total knee arthroplasty and it is believed that total knee arthroplasty is a useful treatment that can resolve arthritis and instability at the same time in older aged patients. Although we were successful with an unconstrained type of TKA, we recommend that if there is severe instability, a constrained type prosthesis should be prepared.

## 4 Conclusion

Reports on the progression of the congenital absence of the ACL in old age are very rare. Since the congenital absence of the ACL in old age could lead to hip and knee osteoarthritis, periodic follow-up is necessary and arthroplasty can be a useful treatment when the osteoarthritis is accompanied by instability.

## Declaration of Competing Interest

Kwang-kyoun Kim, Jae-kyu Choi, Dae-young Kim and Tae-hyeong Kim have nothing to declare.

## Funding

Kwang-kyoun Kim, Jae-kyu Choi, Dae-young Kim and Tae-hyeong Kim have nothing to declare.

## Ethical approval

The patient provided consent for data concerning this case to be submitted for publication and approved by the internal review board of our institution (KYUH 2019-02-001).

## Consent

Written informed consent was obtained from the patient for publication of this case report and accompanying images. I attached a informed consent.

## Author contribution

Kwangk-kyoun Kim contributed of concept, design, data collection, data analysis interpretation, writing the paper.

Jae-kyu Choi, Dae-young Kim and Tae-hyeong Kim contributed in data collection.

## Registration of research studies

This case report is not a research involving human participants.

## Guarantor

Kwangkyoun Kim is the guarantor responsibility for the work and/or the conduct of the study, had access to the data, and controlled the decision to publish.

## Provenance and peer review

Not commissioned, externally peer-reviewed.

## CRediT authorship contribution statement

**Kwang-kyoun Kim:** Writing - original draft. **Tae-hyeong Kim:** Data curation. **Dae-young Kim:** Validation. **Jae-kyu Choi:** Software.
